# Preoperative ultrasound diagnosis of appendiceal diverticulitis in a CT limited setting: A case report

**DOI:** 10.1016/j.radcr.2026.04.034

**Published:** 2026-05-14

**Authors:** Yohannes Girma Zewdie, Moti Negesse Wami, Hana Yeshewas Genetirune

**Affiliations:** a Department of Radiology, Addis Ababa University, Addis Ababa, Ethiopia; b Department of Surgery, Batu General Hospital, Batu, Ethiopia

**Keywords:** Appendiceal diverticulitis, Acute appendicitis mimic, Ultrasound diagnosis, Appendectomy, Diverticular disease of appendix

## Abstract

Appendiceal diverticulitis is a rare inflammatory condition that often mimics acute appendicitis but carries a higher risk of complications. We report a 50-year-old female presenting with chronic right lower quadrant pain for six months without systemic symptoms. Ultrasound revealed a focal diverticular outpouching from the mid appendiceal segment with surrounding inflammation and no fluid collection. In a resource-limited setting, the patient underwent open surgery based on ultrasound findings. The postoperative course was uneventful, and she was discharged on the second day. Intraoperative and histopathologic findings confirmed appendiceal diverticulitis without malignancy. This case highlights the value of ultrasound in diagnosing appendiceal diverticulitis when computed tomography is unavailable.

## Introduction

Appendiceal diverticulitis is a rare inflammatory condition resulting from inflammation of diverticula arising from the appendix, with a reported incidence ranging from 0.004% to 2.1% in appendectomy specimens [1,2]. Clinically, it often mimics acute appendicitis; however, it is associated with a higher risk of perforation, delayed diagnosis, and postoperative complications [3]. Preoperative diagnosis is challenging due to nonspecific clinical features, but imaging particularly ultrasound and computed tomography can demonstrate characteristic findings such as focal diverticular outpouchings with surrounding periappendiceal inflammatory changes [4,5]. Early recognition is important, as appendiceal diverticulitis has been reported to have an association with appendiceal neoplasms, including mucinous tumors and adenocarcinoma [1,6].

## Case presentation

A 50-year-old female presented with chronic right lower quadrant abdominal pain of six months 'duration. The pain was associated with mildly decreased appetite. She denied fever, weight loss, vomiting, or changes in bowel habits. The patient had visited multiple clinics and hospitals and received several courses of antibiotics without symptomatic improvement, likely due to intermittent inflammation of the diverticulum. There was no significant past medical or surgical history.

Her vital signs were within normal limits. On physical examination, there was mild right lower quadrant point tenderness without rebound tenderness. The white blood cell count was normal, while C-reactive protein (CRP) was mildly elevated.

Based on the ultrasound diagnosis of appendiceal diverticulitis, the patient underwent open surgery. The postoperative course was smooth, and she was discharged home on the second postoperative day. She remained asymptomatic during subsequent follow-up visits.

## Imaging findings

**Fig.1:**
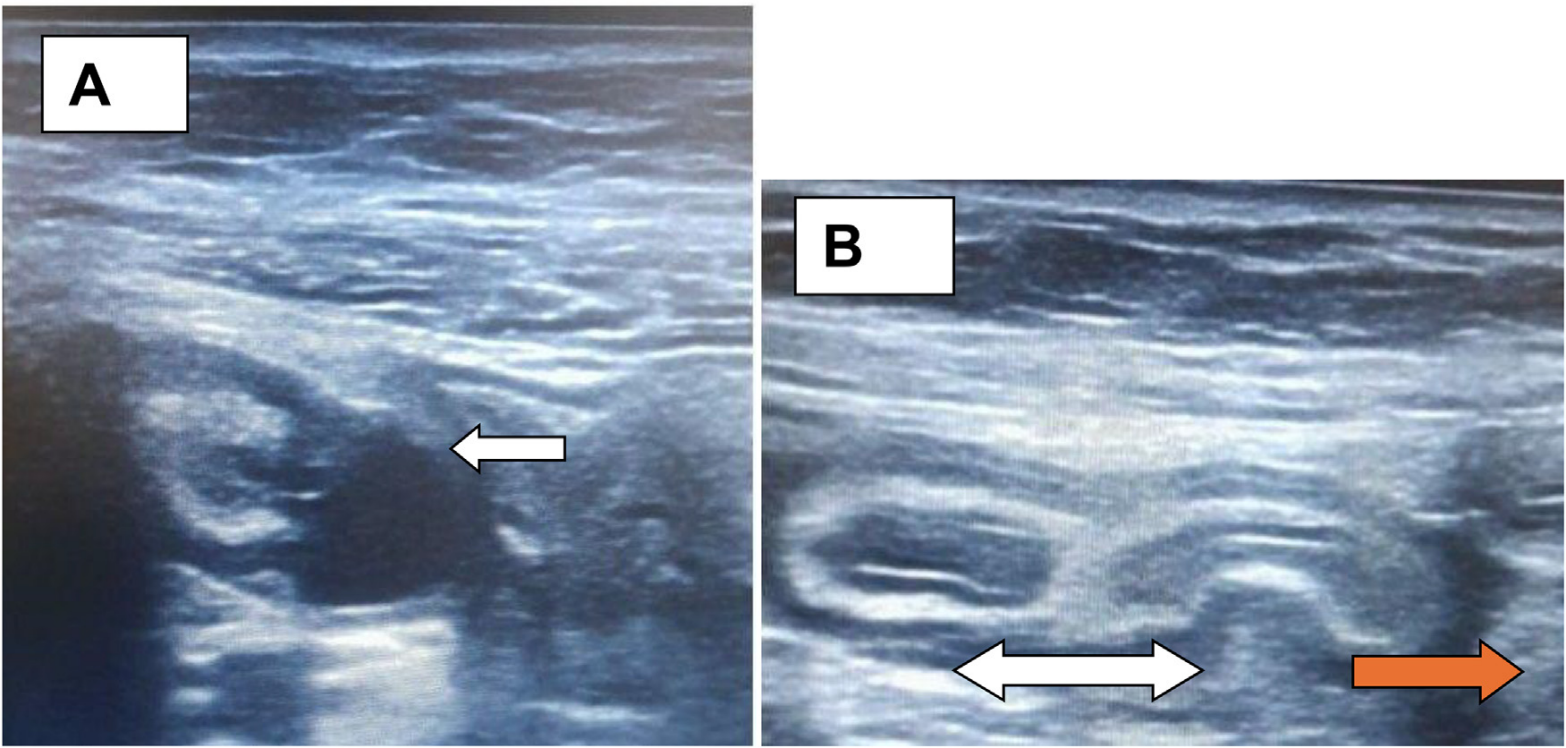
Representative ultrasound images in the transverse (A) and long-axis (B) planes demonstrate an enlarged appendix with focal outpouchings of the appendiceal wall, containing anechoic fluid with posterior acoustic enhancement, consistent with diverticula (arrow). The long-axis view shows appendix (double arrow head) with minimal periappendiceal inflammation (red arrow).

An abdominal ultrasound was performed as the initial imaging modality, with focused evaluation of the right lower quadrant. The appendix was visualized separately and demonstrated a focal outpouching arising from the mid-segment of the appendiceal lumen, consistent with a diverticulum with a hyperemic wall. (Fig. 1A)

The remainder of the appendix appeared normal, with a maximal diameter of 8mm and a single-wall thickness of 3.6 mm. The appendix was compressible, and no fecolith was identified. Surrounding periappendiceal fat appeared inflamed, without evidence of periappendiceal fluid collection or abscess formation. (Fig. 1B)

Given the patient’s lean body habitus and the unavailability of computed tomography, a diagnosis of appendiceal diverticulitis was made based on the ultrasound findings, and surgical management was recommended.

## Discussion

Diverticular disease of the appendix is an uncommon entity and is classified as congenital or acquired, with the acquired from being more prevalent and typically occurring in adults [2,3]. Patients may present with acute or chronic right lower quadrant pain, often without the classic systemic manifestations of acute appendicitis, which can lead to repeated misdiagnosis and delayed treatment [3,7].

Imaging plays a crucial role in establishing a preoperative diagnosis. Ultrasound findings include visualization of the appendix with focal hypoechoic or anechoic outpouchings representing diverticula, associated echogenic periappendiceal fat, and relative preservation of appendiceal luminal diameter compared to acute appendicitis [4,8]. Computed tomography, when available, is considered the imaging modality of choice and typically demonstrates appendiceal wall thickening, diverticula, and periappendiceal inflammatory changes, with or without complications such as perforation or abscess formation [5,9].

**Fig. 2:**
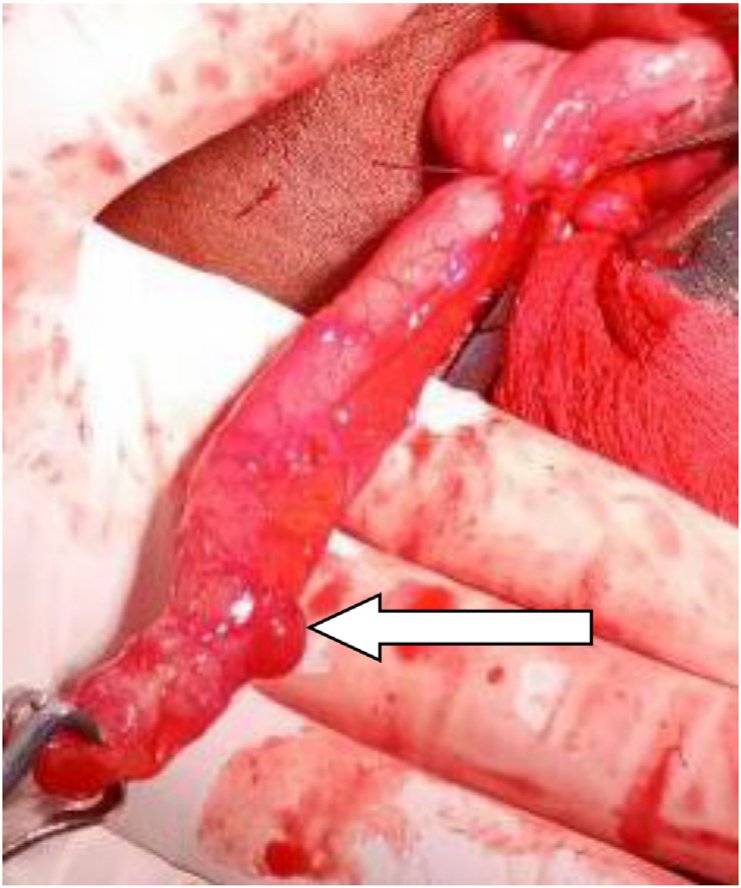
Intraoperative photograph of the appendix demonstrating focal outpouching of the distal appendiceal wall with surrounding hyperemia, suggestive of inflammation. The appendix appears distended.

Appendiceal diverticulitis is clinically significant because it has been reported to have a higher rate of perforation than acute appendicitis and a notable association with underlying appendiceal neoplasms, warranting careful histopathologic evaluation following appendectomy [1,6,10]. In the present case, the chronic nature of symptoms, absence of systemic inflammatory signs, and characteristic ultrasound findings allowed for a preoperative diagnosis despite the lack of CT imaging. Surgical (Fig. 2) and histopathologic findings (sections show an appendiceal diverticulum arising from the appendiceal wall. The diverticular wall demonstrates inflammatory cell infiltration consistent with diverticulitis. The remaining appendiceal mucosa and wall were unremarkable. There was no evidence of perforation. No dysplasia or malignant cells are identified) confirmed appendiceal diverticulitis without evidence of malignancy, and the patient remained asymptomatic on follow-up.

## Conclusion

Appendiceal diverticulitis is an uncommon but important differential diagnosis in patients presenting with right lower quadrant pain. Awareness of its imaging features, particularly on ultrasound in resource-limited settings, can facilitate early diagnosis and appropriate surgical management. Prompt recognition is essential due to the increased risk of perforation and potential association with appendiceal neoplasms.

## Patient consent

Both oral and written informed consent were obtained from the patient for publication of this case report and the associated images.
